# Canalized to heat, plastic to cold: adaptive coordination of leaf and seed strategies in populations spanning an elevational gradient

**DOI:** 10.1111/nph.70912

**Published:** 2026-01-11

**Authors:** Cleber J. N. Chaves, Danilo U. Tavares, Isabella V. Lemos‐Silva, João P. S. P. Bento, Henrique Vilela‐Bianchini, Paulo Aecyo, Tami C. Cacossi, Marília M. Tavares, Gabriel P. Sabino, Vitor de A. Kamimura, Wagner L. dos Santos, Lucas N. Gonçalves, Karina T. Silva, Juliana L. S. Mayer, Rafael V. Ribeiro, Diego Escobar‐Escobar, Kenneth J. Feeley, Clarisse Palma‐Silva

**Affiliations:** ^1^ Laboratory of Evolutionary Ecology and Plant Genomics, Departamento de Biologia Vegetal, Instituto de Biologia Universidade Estadual de Campinas (UNICAMP) Campinas SP 13083‐970 Brazil; ^2^ Department of Biology University of Miami Coral Gables FL 33146 USA; ^3^ Laboratory of Plant Anatomy, Departamento de Biologia Vegetal, Instituto de Biologia Universidade Estadual de Campinas (UNICAMP) Campinas SP 13083‐970 Brazil; ^4^ Postgraduate Program in Plant Biology Instituto de Biologia Universidade Estadual de Campinas (UNICAMP) Campinas SP 13083‐970 Brazil; ^5^ Laboratory of Crop Physiology, Departamento de Biologia Vegetal, Instituto de Biologia Universidade Estadual de Campinas (UNICAMP) Campinas SP 13083‐970 Brazil; ^6^ Grupo de Pesquisa em Tecnologia Ambiental Instituto Tecnológico Vale de Desenvolvimento Sustentável Belém PA 66055‐090 Brazil; ^7^ Fairchild Tropical Botanic Garden Coral Gables FL 33156 USA

**Keywords:** elevational gradient, local adaptation, phenotypic plasticity, seed germination, thermal tolerance, trait canalization, trait network

## Abstract

- In tropical mountains, surviving temperature extremes demands finely tuned strategies. We investigated how populations of the bromeliad *Pitcairnia flammea* across a 2200 m elevational gradient balance genetic canalization and plasticity, and whether thermal strategies are coordinated between seeds and leaves.
- Seven populations (*n* ≥ 20 per site) were studied in the field and in a > 2‐yr common‐garden experiment. Leaf traits (mass per area, area, succulence, stomatal, and trichome densities) and thermal tolerance (*T*_50_ for heat and cold) were measured, and germination assays (10–35°C) quantified seed thermal performances. Multivariate analyses linked leaf and seed traits to elevation and local thermal conditions.
- Heat tolerance and leaf traits were maintained in the common garden, indicating strong canalization, whereas cold tolerance was highly plastic, decreasing by up to 17.9°C. Seeds from high elevations germinated faster, with higher cardinal temperatures and *c*. 230 fewer growing degree days than lowland seeds.
- Thermal niche differentiation in *P. flammea* arises from canalized heat resistance and plastic cold responses, coordinated across leaves and seeds. Considering thermal traits across life stages improves predictions of population resilience under climate warming.

## Resumo

- Em montanhas tropicais, a sobrevivência a extremos térmicos exige estratégias finamente ajustadas. Investigamos como populações da bromélia *Pitcairnia flammea* distribuidas ao longo de um gradiente altitudinal de 2.200 m equilibram canalização genética e plasticidade, e se as estratégias térmicas são coordenadas entre sementes e folhas.
- Sete populações (*n* ≥ 20 por local) foram estudadas em campo e em um experimento *common garden* com duração de mais de dois anos. Foram avaliados caracteres foliares (massa por área, área foliar, suculência, densidades estomática e de tricomas) e a tolerância térmica (T_50_ ao calor e ao frio), além de testes de germinação (10–35°C) para quantificar o desempenho térmico das sementes. Análises multivariadas relacionaram os caracteres foliares e das sementes à elevação e às condições térmicas locais.
- A tolerância ao calor e os caracteres foliares foram mantidos em *common garden*, indicando forte canalização, enquanto a tolerância ao frio mostrou alta plasticidade, reduzindo‐se em até 17,9°C. Sementes de populações de maior altitude germinaram mais rapidamente, apresentaram temperaturas cardinais mais elevadas e necessitaram de aproximadamente 230 graus‐dia a menos do que sementes de populações de baixa altitude.
- A diferenciação do nicho térmico em *P. flammea* resulta da resistência canalizada ao calor e de respostas plásticas ao frio, coordenadas entre folhas e sementes. A consideração de caracteres térmicos ao longo dos diferentes estágios do ciclo de vida aprimora as previsões sobre a resiliência populacional frente ao aquecimento climático.

## Introduction

Natural selection favors traits that maximize fitness under specific environmental conditions (Kawecki & Ebert, 2004). As a result, species spanning heterogeneous environments usually exhibit divergent phenotypes as a product of the interactions between genotype and environment. This phenotypic divergence may stem from genetically based local adaptations or from the variable expression of the same genotypes under distinct conditions (i.e. phenotypic plasticity). Local adaptation likely arises when gene flow among populations is limited, increasing the chances that adaptive mutations become fixed, whereas phenotypic plasticity is more likely to evolve when there is extensive gene flow (Scheiner, 2013; Palacio‐López *et al*., 2015). However, local adaptation and phenotypic plasticity are not mutually exclusive and may give rise to one another (e.g. Pigliucci *et al*., 2006; Wang & Zhou, 2022).

Phenotypic plasticity is often favored in heterogeneous or highly seasonal environments, such as those at high latitudes and elevations (Humboldt, 1807; Wallace, 1878; Dobzhansky, 1950). While some genotypes express different phenotypes across environments, others remain canalized, showing little variation despite environmental or genetic changes (Waddington, 1942; Flatt, 2005). Traits under strong stabilizing selection, particularly in high‐stress conditions, may be especially prone to canalization (Wagner *et al*., 1997; Flatt, 2005). Despite the phenotypic stasis, canalization can mask cryptic genetic variation (CGV), which can be revealed under novel or relaxed conditions (‘decanalization’), increasing phenotypic diversity (Flatt, 2005; Schlichting, 2008; Iwasaki *et al*., 2013; Wang & Zhou, 2022). In turn, plasticity can lead to genetic assimilation, where specific environmentally induced phenotypes become fixed under new selective pressures (Waddington, 1942; Pigliucci *et al*., 2006; Lande, 2009; Levis & Pfennig, 2019; García‐Verdugo *et al*., 2023). Distinguishing whether population divergence arises from plasticity, canalization, or genetic assimilation is key to understanding plant adaptation across temporal and spatial environmental gradients.

Evaluating whether phenotypes are canalized or plastic can be challenging. For instance, buffering morphology variance across diverse environmental conditions often requires higher variation in gene expression, through epistatic interactions, and physiological adjustments (Hermisson & Wagner, 2004; Flatt, 2005; Schlichting, 2008). Phenotypic plasticity itself can be divided into morphological and physiological plasticity (Bradshaw, 1965; Hou *et al*., 2015; Marchiori *et al*., 2017). Physiological plasticity is generally less costly, potentially occurring over short time periods and across various tissues, whereas morphological plasticity demands structural changes, often involving tissue replacement (Bradshaw, 1965; Grime & Mackey, 2002; Hou *et al*., 2015). Additionally, traits are often part of phenotypic integration, which refers to sets of tightly correlated traits shaped by trade‐offs and developmental constraints (Wagner *et al*., 2007; Klingenberg, 2009; Palow *et al*., 2012; Damián *et al*., 2018). Therefore, selective pressures on plasticity or canalization should affect multiple traits and ontogenetic stages.

In plants, as sessile organisms, adaptations to environmental changes are crucial for survival. The effects of temperature shifts, for instance, range from changes in membrane fluidity and protein folding to disruptions in plant growth, phenology, and seed germination (see Hasanuzzaman, 2020; Lamers *et al*., 2020). As such, thermal adaptations are inherently multitrait, encompassing both thermal tolerances and the ability to regulate temperature as a buffering or avoidance mechanism (Hasanuzzaman, 2020; Lamers *et al*., 2020; Chaves *et al*., 2024). Germination, as one of the earliest life‐stage transitions, sets the environmental context for seedling establishment and influences the expression and selection of many postgermination traits (Donohue *et al*., 2010; Cochrane *et al*., 2011; Fernández‐Pascual *et al*., 2019; Hirst *et al*., 2025). Because of this linkage, both plastic responses and genetically based adaptations to temperature often arise from coordinated trait combinations rather than isolated traits. Understanding plant adaptation to thermal variation requires an integrative approach that considers the interplay between plasticity and local adaptation across an integrated perspective on the plasticity of germination and postgermination phenotypes, as well as on how closely these traits are coordinated across thermal gradients.

**Fig 1:**
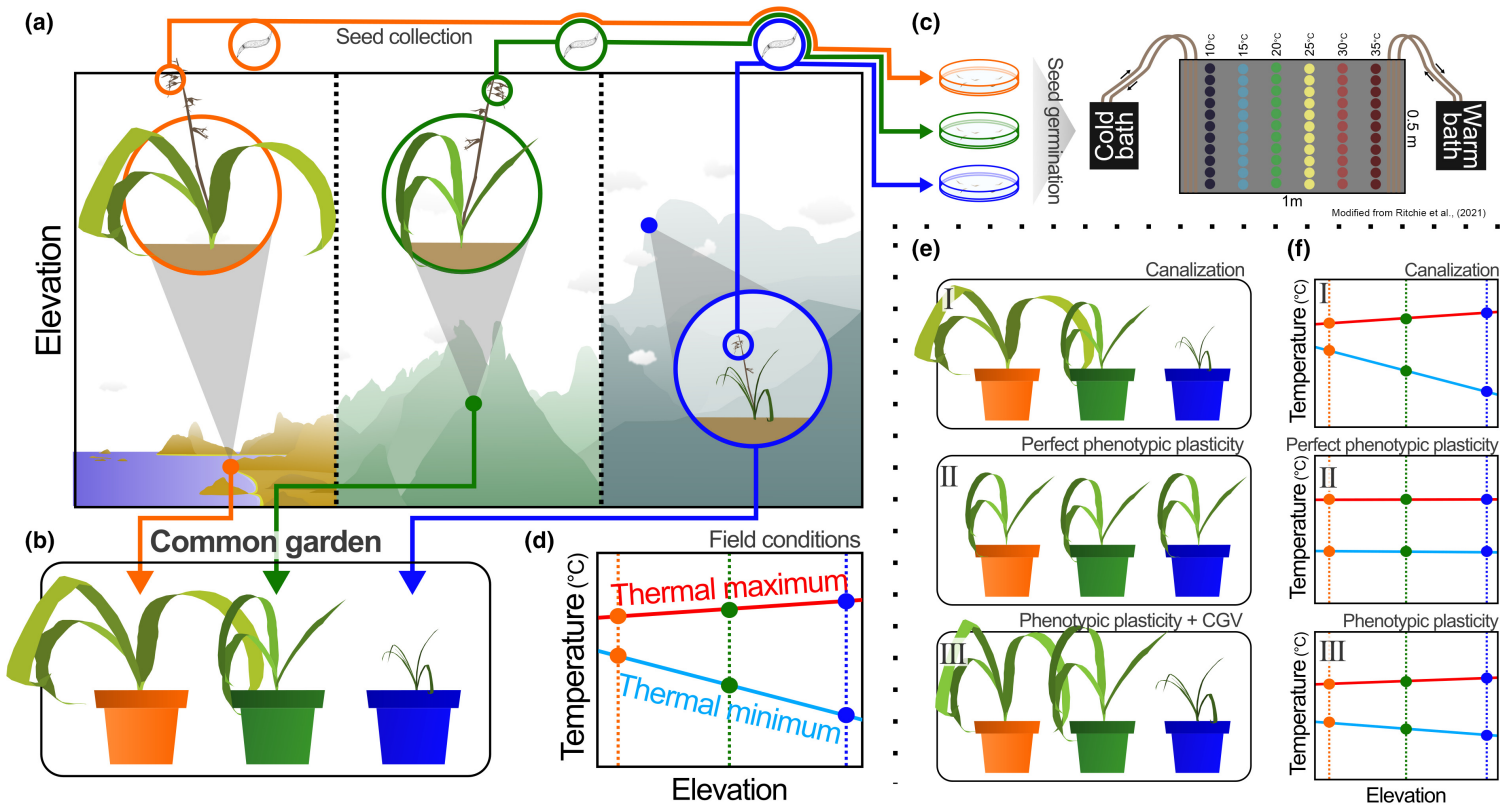
Overall methodology (a–f) and hypotheses for testing plasticity and canalization in *Pitcairnia flammea* (Bromeliaceae) thermal adaptations across elevations. From plants sampled along a > 2200 m elevation gradient, we compared plant morphology and thermal tolerance in field‐sampled individuals (a) and those grown in a common garden (b). To compare thermal strategies in plants and seeds, we collected seeds from the same populations and tested germination along a thermal gradient (10–35°C) generated by an aluminum plate connected to two water baths (c; see Supporting Information Fig. S2 for more details). These experiments allowed us to determine thermal limits for photochemistry and germination (d) and to assess how morphology (e) and thermal performances (f) shift under common‐garden conditions. Cryptic genetic variation (CGV of traits) adds hidden variation that can act alongside the phenotypic plasticity of any trait.

Thermal adaptations of plants across latitudes and elevations are well documented (e.g. Cuesta *et al*., 2020; Lancaster & Humphreys, 2020; Chaves *et al*., 2025). A recent study has shown that such adaptations can also occur among populations of the same species, as in *Pitcairnia flammea* Lindl. (Bromeliaceae), where a trade‐off exists between thermal tolerance and thermal avoidance (Chaves *et al*., 2024). Here, we examine the thermal strategies of *P. flammea* populations along a broad elevation gradient to distinguish local adaptations from phenotypic plasticity and link these strategies to seed germination behavior (Fig. 1). Specifically, we asked: (1) To what extent are population‐level thermal strategies shaped by plasticity vs canalization? (2) How do physiological and morphological adaptations potentially differ in their responses to thermal gradients? (3) To what extent do leaves and seeds share similar thermal strategies? We hypothesized that Photosystem II thermal tolerance would exhibit greater plasticity, whereas morphological traits would show lower variability, being more tightly associated with population origin and seed thermal behavior. To address these questions, we analyzed *P. flammea* individuals sampled along an elevational gradient of > 2200 m above sea level (asl) in Brazil's Atlantic Coastal Rainforest, both in the field (Fig. 1a) and under common‐garden conditions (Fig. 1b). We further investigated thermal germination behavior at the population level using a novel aluminum plate method to generate a controlled thermal gradient (Fig. 1c). By integrating data on seed germination with assessments of plant morphological (Fig. 1e) and physiological traits (Fig. 1f) across contrasting environments, this study seeks to elucidate how plasticity and adaptation interact to shape plant thermal strategies across life stages and environmental gradients.

## Materials and Methods

### Sampling and common‐garden cultivation

To assess whether differences in thermal strategies – including thermal tolerance and leaf traits – among *Pitcairnia flammea* populations are driven by phenotypic plasticity and/or genetic canalization, we cultivated ≥ 20 individuals per population from seven sites spanning an elevation gradient from sea level to 2200 m asl (Supporting Information Fig. S1). Plants were grown individually in pots filled with a 1 : 1 mixture of organic soil and expanded clay pellets, randomly arranged, and maintained under uniform glasshouse conditions with sprinkler irrigation applied for 5 min, five times daily, for over 2 yr before trait measurements (to be described later). During this period, the average temperature was 19°C, relative humidity averaged 57.7%, and the maximum photosynthetic photon flux density (PAR) was *c*. 500 μmol m^−2^ s^−1^.

### Thermal tolerance and leaf traits estimation

To quantify morphological and physiological adaptations to thermal variation along the elevational gradient, we assessed photosynthetic efficiency (Chla fluorescence) across a range of experimental temperatures as a physiological indicator of heat and cold tolerance. Morphological traits included leaf mass per area (LMA), succulence index (SI; Ogburn & Edwards, 2010), leaf area (LA, measured using the Petiole Pro app, v.23.11.8), stomatal density, and trichome density on both abaxial and adaxial leaf surfaces. For stomatal and trichome quantification, leaf fragments were preserved in neutral buffered formalin, dehydrated in ethanol, cleared with a hydrogen peroxide–glacial acetic acid solution, stained with safranin, and mounted in glycerol following a modified Franklin (1945) protocol; images were captured with an Olympus BX51 microscope and analyzed in imagej (Scheiner, 2013). Physiological thermal tolerance was measured by exposing dark‐adapted leaf disks to progressively increasing temperatures (27–60°C; 1°C every 3 min, with *F*_v_/*F*_m_ recorded every 3°C) or decreasing temperatures (20 to −22°C at the same rate, with measurements every 5°C). Samples were held at target temperatures for *c*. 1 min before each reading to equalize leaf temperature and ensure ≥ 10 min between measurements. Temperature control was maintained using an ultra‐thermostatic water bath (2050; Thoth, Sao Paula, Brazil), and K‐type thermocouples attached to a subset of samples verified leaf temperature accuracy (TH‐096; Instrutherm, Sao Paula, Brazil). *F*_v_/*F*_m_ was measured using a PAR‐FluorPen FP 110/D (PSI Instruments, Drasov, Czech Republic) in the field and an FMS1 fluorometer (Hansatech, King's Lynn, UK) under controlled conditions. Heat and cold tolerance were defined as the temperature causing a 50% decline in *F*_v_/*F*_m_ (*T*_50_), estimated by fitting sigmoidal curves with the ‘drc’ R package (Ritz *et al*., 2015), following Knight & Ackerly (2003), Gimeno *et al*. (2009), and Godoy *et al*. (2011). Measurements were taken after sunrise (cold tolerance) and shortly after noon (heat tolerance) to capture daily thermal extremes. Common‐garden assessments followed a 3‐d acclimation period under a 12‐h photoperiod at 15°C for cold and 30°C for heat, with additional methodological details provided in Chaves *et al*. (2024).

### Seed germination tests

To estimate the thermal performance of *P. flammea* seeds from across the elevational gradient, we collected fruits from six of the seven sampled populations (fruits were unavailable at the highest elevation site). After collection, seeds were stored for up to 2 months at room temperature in ziplock bags containing silica to prevent fungal growth and loss of seed viability. Germination tests were performed at six constant temperatures (10, 15, 20, 25, 30, and 35°C) using a thermal gradient plate, adapted from Ritchie *et al*. (2021). The device operates by circulating hot and cold water through an aluminum plate equipped with embedded sensors, enabling precise temperature control under constant light conditions (Figs 1c, S2). For each population and temperature, four replicates of 20 seeds were placed in Petri dishes lined with double layers of filter paper and continuously supplied with distilled water. At the highest temperatures (30 and 35°C), evaporation was too rapid, causing water droplets to accumulate on the lid and leaving the filter paper dry. In these cases, seeds were floated directly on distilled water, a method previously shown not to affect embryo viability and consistent with the species' ability to germinate on water surfaces (Chaves *et al*., 2025). In total, 114 dishes and 480 seeds were tested. Germination was recorded every 2 d over a 60‐d period, considering seeds as being germinated when the hypocotyl exceeded one‐third of the seed length (*c*. 0.5 mm). At the end of the experiment, nongerminated seeds were tested for viability using tetrazolium staining (Ministério da Agricultura e da Reforma Agrária, 2009) and examined under a stereoscope. The nonviable seeds were removed from the final proportion results.

### Statistical analysis

To estimate whether thermal strategies are shaped by plasticity or canalization, we compared the thermal tolerance and the morphological leaf traits measured in the field and under common‐garden conditions across populations. To test the differences between both treatments, we used *t*‐tests with a 95% confidence interval. To compare the field and common‐garden tendencies across environments, we used regression slope comparisons using the ‘multcomp’ and ‘emmeans’ R packages (Piepho, 2004; Lenth, 2024), modeling each trait as a function of elevation, mean annual temperature (Fick & Hijmans, 2017), and heating degree days (HDD; Mistry, 2019a,b). HDD was adopted as a metric to quantify the duration and extent to which temperatures remain above a base threshold (18°C, the highest temperature adopted by Mistry, 2019a,b), capturing both temperature extremes and local variations in heating demand driven by insulation, solar exposure, wind, and humidity.

To reduce trait dimensionality and identify patterns of variation in morphological and physiological thermal strategies across environments and elevations, we used a principal component analysis (PCA) using the factominer package (Lê *et al*., 2008). To address missing trait values, we used a regularized iterative PCA for imputation, based on the first two principal components, using the missmda package (Josse & Husson, 2016). To assess whether the previously reported trade‐off between thermal tolerance and avoidance traits (Chaves *et al*., 2024) varies between field and common‐garden conditions, we used Pearson correlation networks, visualized using alluvial diagrams generated with the ggalluvial package (Brunson & Read, 2023), comparing correlations between *T*_50_ (heat and cold) and morphological traits under both settings.

To assess seed thermal performances, we adopted the framework from Parmoon *et al*. (2015) to estimate the cardinal temperatures for germination: base (*T*_b_), optimum (*T*_o_), and ceiling (*T*_c_). We modeled cumulative germination over time at each temperature (10, 15, 20, 25, 30, and 35°C) using a four‐parameter log‐logistic model implemented via the ‘drm’ function in the ‘drc’ R package (Ritz *et al*., 2015). From each fitted model, we extracted the time to 50% germination (*D*_50_). We calculated the germination rate as the inverse of the time to 50% germination (*r*_50_ = 1/*D*_50_), and modeled it as a quadratic function of temperature to derive the cardinal temperatures analytically. To estimate the thermal time requirement for germination, we adopted the growing degree day (GDD) approach, calculated as GDD = *D*_50_ × (*T*−*T*_b_), where *T* is the incubation temperature (Golmohammadzadeh *et al*., 2022).

To test whether seed thermal performances align with leaves' thermal strategies, we first tested how cardinal temperatures and GDD relate to local environments, using linear and quadratic regression models against elevation, mean annual temperature, and HDD. We evaluated model performance based on *R* ^2^ and *P*‐values. Finally, we used Pearson correlation analysis to examine the relationship between seed‐derived traits (*T*_b_, *T*_o_, *T*_c_, and GDD) and thermal tolerance, as well as morphological traits from both the field and the common garden. These correlations were visualized using heatmaps to highlight consistent or divergent patterns across life stages.

## Results

### Field vs common‐garden comparisons

**Fig 2:**
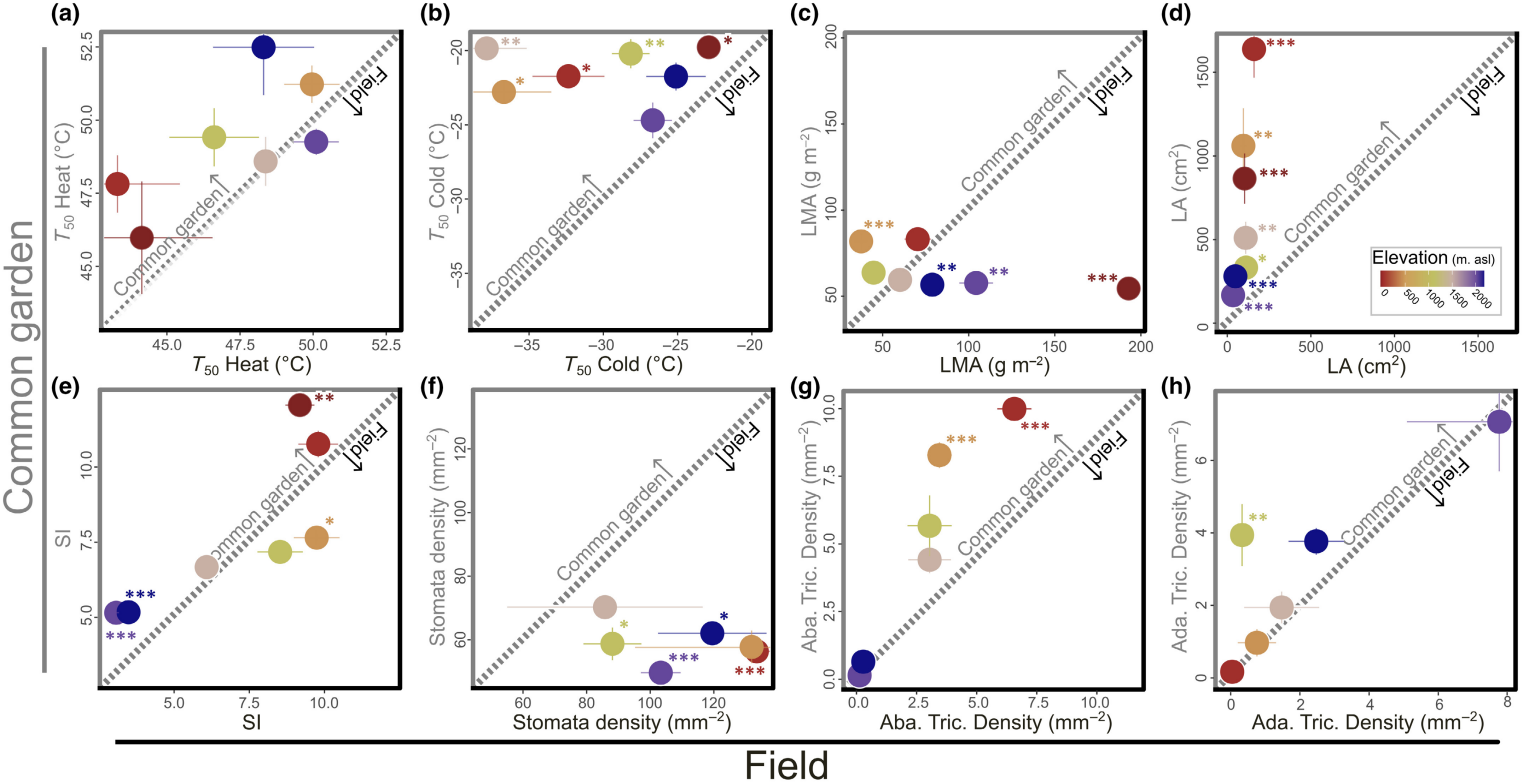
Comparison between thermal tolerance (a, b) and leaf traits (c–h) between field‐sampled (*x*‐axis) and common‐garden (*y*‐axis) plants from seven *Pitcairnia flammea* populations spanning an elevation gradient of > 2200 m. Warm to cool colors represent the populations' elevation of origin (from low to high). Error bars represent SE. Significant differences (*t*‐test) between traits from field‐sampled and common‐garden plants are indicated by *, *P* < 0.05; **, *P* < 0.01; ***, *P* < 0.001. The diagonal line represents the 1 : 1 relationship, with deviations indicating shifts between treatments. *T*_50_ Heat and Cold: maximum and minimum temperature, specifically causing a 50% decline in Chl *F*_v_/*F*_m_. Ada. and Aba. Trich. density, adaxial and abaxial trichome densities; LA, leaf area; LMA, leaf mass per area; SI, succulence index.

We detected significant differences between field and glasshouse measures of all traits except heat tolerance (Fig. 2). Heat tolerance remained consistent across all populations (Fig. 2a), while cold tolerance varied between environments, with only highland plants showing no significant change (Fig. 2b). *T*_50_ cold values differed by as little as 1.9°C in highland plants and up to 17.9°C in one midland population. Morphological traits exhibited a range of patterns: adaxial trichome density showed minimal differences except in one midland population (Fig. 2h); stomatal density was generally higher in field plants (Fig. 2f); LMA displayed distinct shifts in each population (Fig. 2c); and LA was consistently larger in glasshouse plants across all populations (Fig. 2d). Notably, glasshouse plants developed leaves ranging from 128 cm^2^ (highland) to nearly 1475 cm^2^ broader (lowland) than those of their field counterparts, highlighting the substantial plasticity of this trait (Fig. 2d).

**Fig. 3:**
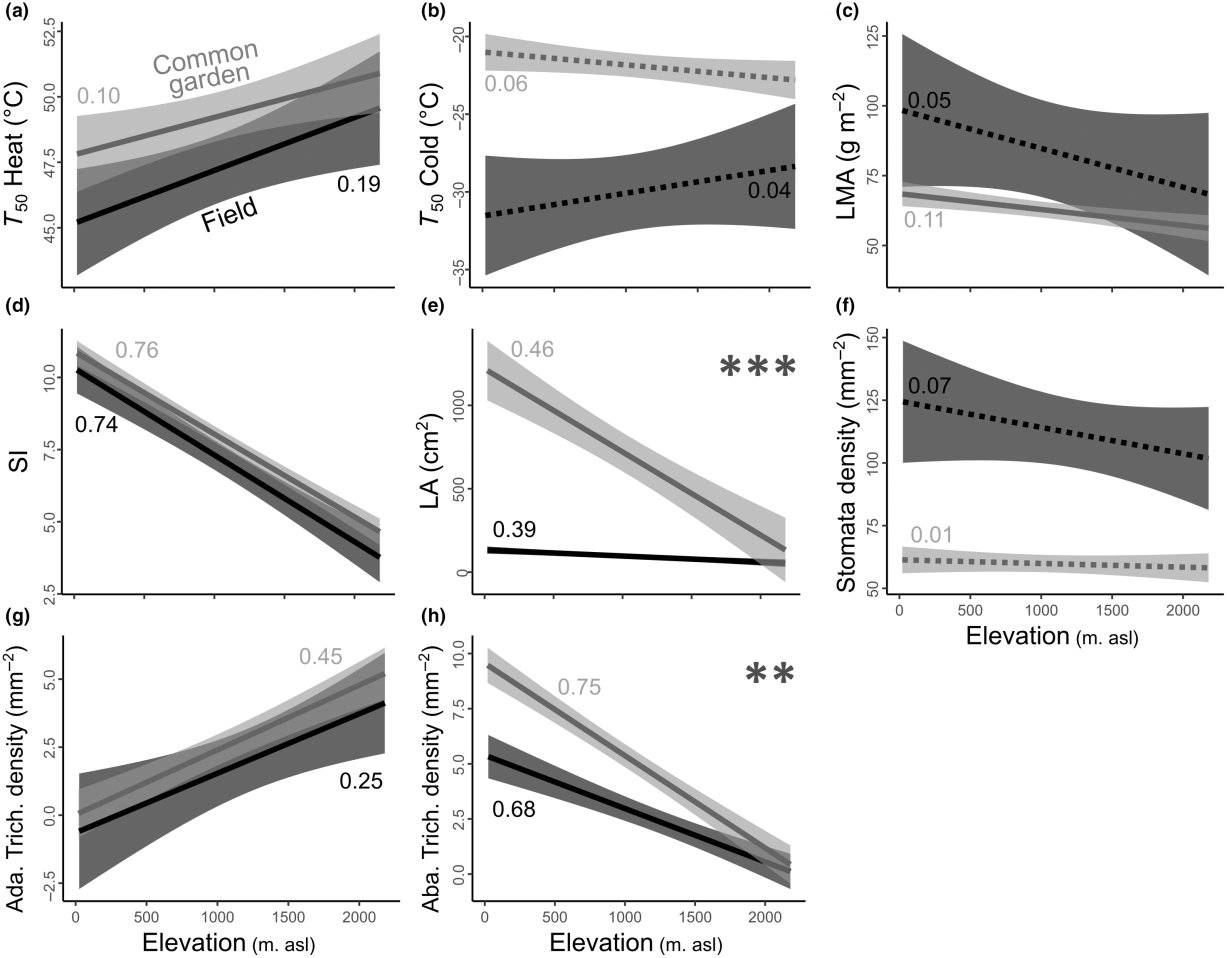
Linear relationships between thermal tolerance (a, b) and leaf traits (c–h) of *Pitcairnia flammea* individuals with elevation shifts. Black and gray lines (with shaded 95% confidence intervals) represent field‐sampled and common‐garden plants, respectively. Numbers indicate the *R* ^2^ values of the regressions. Significant differences between slopes (*t*‐test) are denoted by *, *P* < 0.05; **, *P* < 0.01; ***, *P* < 0.001. Dashed lines indicate no significant relationships. *T*_50_ Heat and Cold: maximum and minimum temperature, specifically causing a 50% decline in Chl *F*_v_/*F*_m_. Ada. and Aba. Trich. density, adaxial and abaxial trichome densities; LA, leaf area; LMA, leaf mass per area; SI, succulence index.

In the field, *P. flammea* exhibited only a modest rise in heat tolerance across elevations, with cold tolerance remaining stable (Fig. 3a,b). High‐elevation populations displayed denser adaxial trichomes alongside reductions in leaf SI, abaxial trichome density, LMA, and LA (Fig. 3). In the common garden, these same trait–elevation trends persisted but with significantly steeper slopes (*P* < 0.05) for LA and abaxial trichomes (Fig. 3e,h). When examined in relation to local thermal environments, traits that strongly aligned with elevation showed inverted relationships with annual mean temperature and, in some cases, diverged from expectations based on HDD (Fig. S3). Specifically, field‐grown plants from warmer, low‐HDD sites exhibited higher cold tolerance – an effect that did not persist under common‐garden conditions (Fig. S3D). Likewise, populations originating from high‐HDD environments showed the highest LMA *in situ*, yet this relationship was completely reversed in the glasshouse (Fig. S3N).

### Changes in thermal strategies

**Fig. 4:**
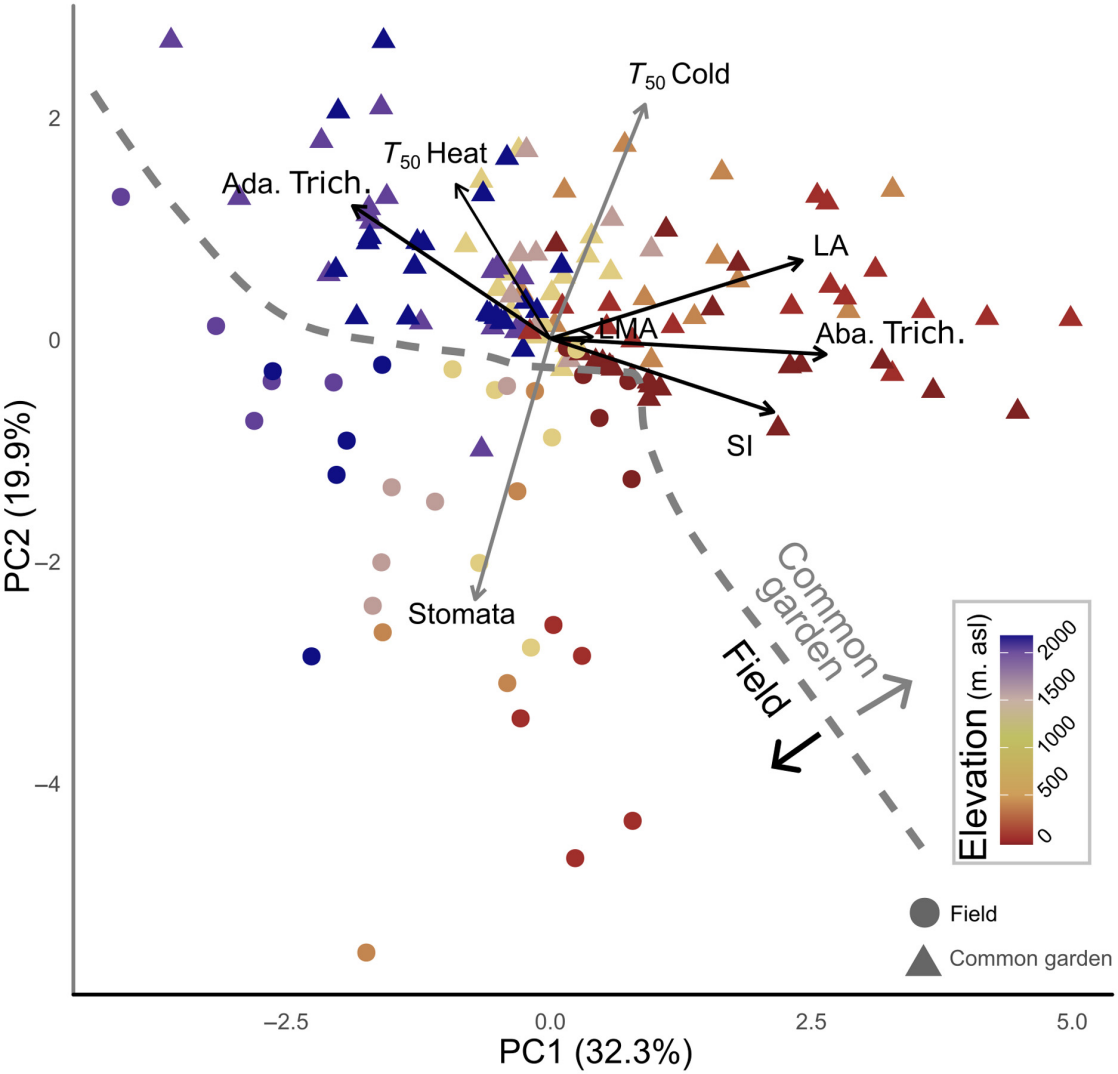
Principal component analysis (PCA) of all sampled *Pitcairnia flammea* individuals by origin (circles: field‐sampled; triangles: common‐garden). Warm to cold colors indicate populations from low to high elevations. Arrows represent the correlations of each trait with the principal components. Black and gray arrows indicate variables more strongly related to the first and second principal components, respectively. The dashed line indicates the division observed between field‐sampled and common‐garden individuals. *T*_50_ Heat and Cold: maximum and minimum temperature, specifically causing a 50% decline in Chl *F*_v_/*F*_m_. Ada. and Aba. Trich., adaxial and abaxial trichome densities; LA, leaf area; LMA, leaf mass per area; SI, succulence index; Stomata, abaxial stomata density.

**Fig. 5:**
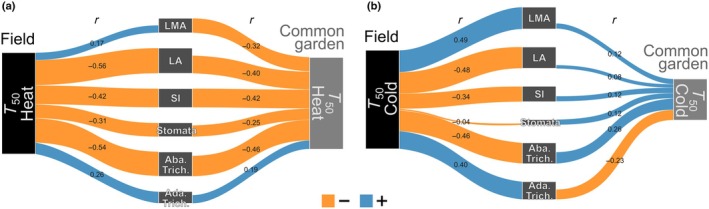
Pearson correlation networks showing relationships between heat (a) and cold (b) tolerances and morphological traits (rectangles in the center) in *Pitcairnia flammea*. Networks are split to compare correlations in field data (left, black rectangles) vs common‐garden data (right, gray rectangles). Values on the links indicate absolute Pearson's *r*; blue links denote positive correlations, orange links negative correlations. *T*_50_ Heat and Cold: maximum and minimum temperature, specifically causing a 50% decline in Chl *F*_v_/*F*_m_. Ada. and Aba. Trich., adaxial and abaxial trichome densities; LA, leaf area; LMA, leaf mass per area; SI, succulence index; Stomata, abaxial stomata density.

The first two components of the PCA explained 52.13% of variation in thermal tolerance and morphological leaf traits (Fig. 4). The first principal component (PC1) was primarily driven by abaxial trichome density, LA, SI, and adaxial trichome density (*r* = 0.88, 0.80, 0.71, and −0.63, respectively) and closely tracked the elevation gradient, separating lowland from highland individuals. The second principal component (PC2), in turn, was primarily shaped by stomatal density and cold tolerance (*T*_50_ cold; *r* = −0.78 and 0.70), effectively distinguishing plants grown in the field from those in the common garden, particularly among lowland populations. These multivariate patterns were reflected in the trait correlation networks (Fig. 5), revealing contrasting patterns for heat and cold tolerance across environments. In the field, *T*_50_ heat exhibited moderate‐to‐strong negative correlations with most morphological traits, particularly with LA (*r* = −0.56), abaxial trichome density (*r* = −0.54), and SI (*r* = −0.42). These relationships persisted, although they attenuated under common‐garden conditions (*r* = −0.40, −0.46, and −0.42, respectively; Fig. 5a). By contrast, *T*_50_ cold showed weaker correlations with morphological traits and notably shifted in both strength and direction between environments (Fig. 5b). In particular, the strongest field correlation of *T*_50_ cold with LMA (*r* = 0.49) weakened considerably in the common garden (*r* = 0.12), although it retained its positive sign (Fig. 5b).

### Thermal seed performances

**Fig. 6:**
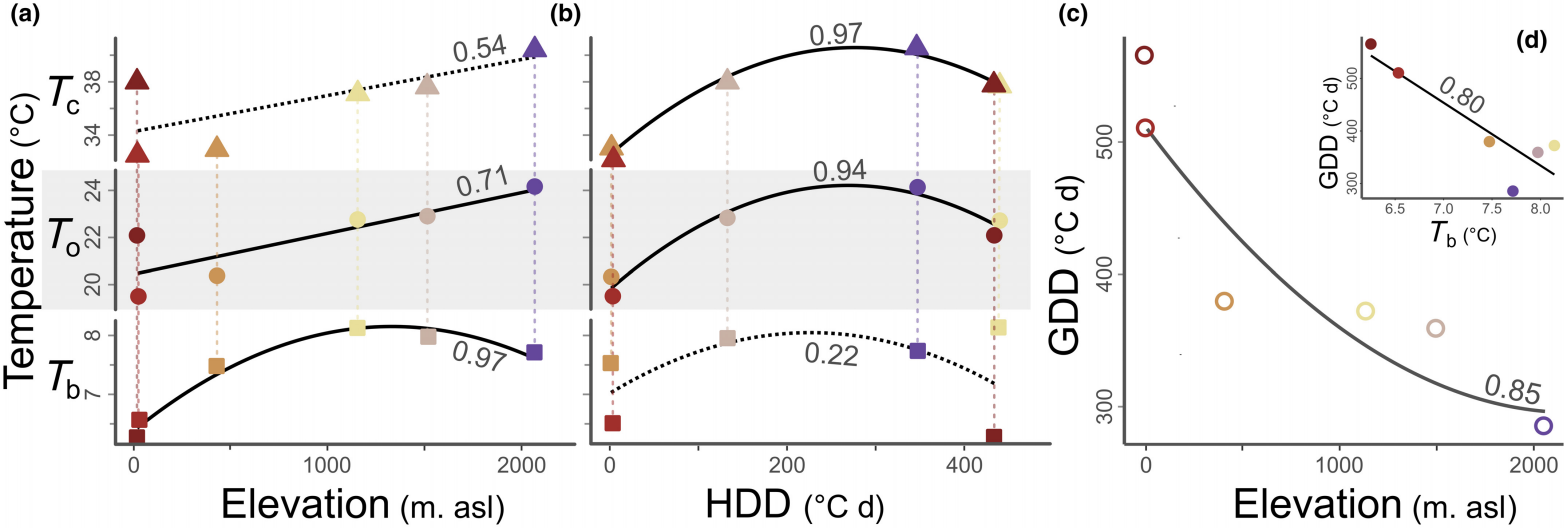
Cardinal temperatures – basal (*T*_b_; square), optimal (*T*_o_; circle), and ceiling (*T*_c_; triangle) – for *Pitcairnia flammea* seed germination in relation to elevation (a) and heating degree days (HDD; b). (c) displays growing degree days (GDD) for seed germination across elevations, while (d) illustrates their relationship with *T*_b_. Numbers indicate the *R* ^2^ values of the regressions. In (a, b), the light gray rectangle separates the regression values for *T*_o_ from those for *T*_c_ and *T*_b_.

Temperature had distinct effects on germination timing and success across the different populations (Figs S4, S5), with 20°C showing the least variation. Differences between extremes of time for the germination of 50% of seeds (D_50_) ranged from 12.5 d under 10°C, reduced to 6.4 d under 20°C, and increased to > 36 d under 30°C (Fig. S5). Highland seeds showed the fastest germination, particularly under the highest temperatures (Figs S4–S5). All cardinal temperatures – basal (*T*_b_), optimal (*T*_o_), and ceiling (*T*_c_)—increased with elevation and HDD (Fig. 6a,b). *T*_b_ showed the narrowest range (6.3 to 8.1°C), while *T*_c_ varied the most (32.5 to 40.6°C), and *T*_o_ range from 19.5 to 24.2°C. *T*_b_ was more strongly associated with elevation, whereas *T*_o_ and *T*_c_ aligned more closely with HDD (Fig. 6a,b). Moreover, GDD decreased significantly with elevation and were negatively correlated with *T*_b_ (Fig. 6c,d; *P* < 0.05). Overall, highland seeds required *c*. 230 GDD less to germinate than lowland seeds, despite having a *T*_b_ *c*. 2°C higher.

### Thermal seed performances vs leaf traits

**Fig. 7:**
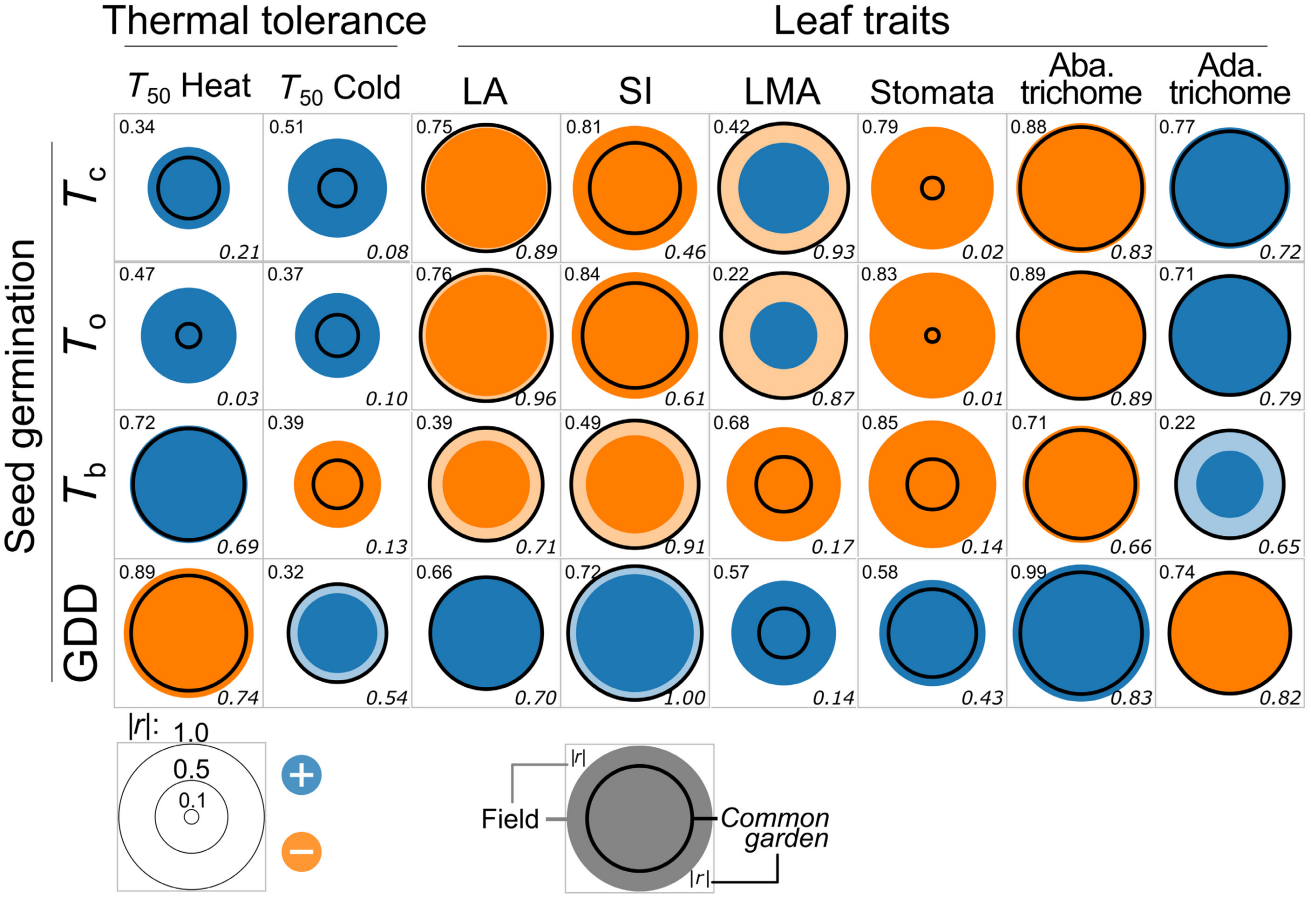
Matrix of Pearson correlations between *Pitcairnia flammea* seed‐derived traits (*T*_b_, *T*_o_, *T*_c_, GDD; rows) and leaf thermal tolerance and morphological traits (columns). Circle size represents the absolute Pearson's *r* value, with blue indicating positive correlations and orange indicating negative correlations. Values in the top left of each cell correspond to field data (circle without outline), while values in the bottom right correspond to common‐garden data (circle with black outline). *T*_50_ Heat and Cold: maximum and minimum temperature, specifically causing a 50% decline in Chl *F*_v_/*F*_m_. Ada. and Aba. trichome, adaxial and abaxial trichome densities; LA, leaf area; LMA, leaf mass per area; SI, succulence index; Stomata, abaxial stomata density.

Overall, thermal seed performances were more strongly associated with heat tolerance than with cold tolerance, particularly under field conditions (Fig. 7). *T*_50_ heat showed a positive correlation with cardinal temperatures – especially with base temperature (*T*_b_; *r* = 0.72) – and a negative correlation with GDD (*r* = −0.89). These correlations were slightly weaker under common‐garden conditions (*r* = 0.69 and −0.74, respectively; Fig. 7). Even stronger correlations were observed between seed traits and leaf morphological traits (Fig. 7), particularly the most associated with heat tolerance – such as LA, SI, stomatal density, and abaxial trichome density (Fig. 5) – with Spearman's *r* values reaching up to 1.0. In general, seeds from lower elevations exhibited colder thermal limits, while also displaying traits associated with lower heat tolerance, and required greater heat accumulation to germinate (i.e. slower germination rates). Plants from these populations displayed broader leaves with water‐saving traits that enhance heat absorption, including high SI and abaxial trichome densities, along with low adaxial trichome density. By contrast, correlations with *T*_50_ cold were weaker and showed opposite trends (*r* = −0.39 with *T*_b_, *r* = 0.32 with GDD) compared with *T*_50_ heat.

## Discussion

This study demonstrates that the thermal strategy of *Pitcairnia flammea* across elevations is shaped by both canalized and plastic local adaptations. While there is a clear trade‐off between tolerance and avoidance strategies, consistent with previous findings (Chaves *et al*., 2024), the traits underlying population‐level differences vary in degree of canalization and plasticity. Differences in heat tolerance and its associated morphological traits across populations tended to remain consistent in the common‐garden setting, suggesting a strong genetic basis. By contrast, cold tolerance exhibited high plasticity, reflecting a loss of tolerance capacity when plants were grown under common‐garden conditions compared with the field. Adaptations to high temperatures are also reflected in population‐level differences in seed thermal performances, particularly in basal germination temperature and accumulated degree days for germination.

### Plasticity and canalization of leaf thermal strategies

Our study reveals differences in heat and cold tolerance in *P. flammea* plants under natural conditions compared with those grown in a common‐garden setting. While the positive relationship between heat tolerance and elevation (and its negative relationship with mean annual temperature) persisted under controlled common‐garden conditions, cold tolerance showed a sharp reduction – up to 18°C – in the common‐garden environment. Considering local HDD, cold tolerance tended to increase in warmer, low‐HDD regions – a pattern not captured by mean annual temperature alone – but this relationship vanished under common‐garden conditions, indicating substantial phenotypic plasticity. Indeed, cold tolerance often showed greater variability across species and stronger associations with local environments and thermal gradients, compared with heat tolerance (e.g. Lancaster & Humphreys, 2020; Chaves *et al*., 2024). Moreover, natural acclimation and exposure to multiple and sequential stressors in the wild can improve physiological tolerances in plants, particularly to cold (Liu *et al*., 2017; Hossain *et al*., 2018; Baier *et al*., 2019). Our findings suggest that the stronger response of cold tolerance to environmental variation likely reflects its inherently higher plasticity rather than genetically based adaptations.

Differences in canalization and plasticity shape not only the heat and cold tolerance, but also the overall thermal strategies of *P. flammea*. Part of the strong canalization observed in heat tolerance may reflect a physiological ceiling imposed by limited capacity for membrane fatty‐acid remodeling and repair (e.g. Yamamoto, 2016; Posch *et al*., 2022; Prasertthai *et al*., 2022). However, negative associations with morphological traits – such as leaf succulence, LA, and stomatal and abaxial trichome densities – were largely maintained under common‐garden conditions, suggesting consistent canalization in water‐holding and heat‐avoidance strategies across populations (Griffiths & Males, 2017; Males, 2017; Midolo *et al*., 2019; Chaves *et al*., 2024). By contrast, while these traits are also related to cold tolerance in the field (with an opposite tendency slope), their relationship weakened or reversed in common‐garden conditions, highlighting the greater plasticity of cold tolerance. This pattern supports that morphological traits tend to be more canalized, whereas physiological traits – here defined specifically as thermal tolerance parameters (*T*_50_ for heat and cold) – generally display greater plasticity in response to environmental changes (Cordell *et al*., 1998; Delagrange *et al*., 2006; Hou *et al*., 2015). Therefore, the maintenance of linkage between morphological traits and heat tolerance in common‐garden contributes to the high canalization of heat tolerance, while the disruption between morphological traits and cold tolerance in the common garden suggests that cold tolerance is a labile trait reflecting physiological plasticity.

Beyond thermal tolerance, the consistent expression of traits, such as stomatal and adaxial trichome densities across both field and common‐garden conditions, supports a history of local adaptation in *P. flammea*. By contrast, shifts in traits like LA, LMA, and abaxial trichome density under controlled conditions highlight phenotypic plasticity and the expression of CGV, as it is not expressed under field conditions (Schlichting, 2008). The particularly strong increase in LA in lowland populations suggests that environmental stress in natural settings may mask underlying genetic variation, especially in warmer sites. The expression of this hidden variation under favorable conditions supports the role of CGV in shaping morphological responses (Iwasaki *et al*., 2013; Cui *et al*., 2018; Donnelly *et al*., 2018; Archambeau *et al*., 2023). This is especially relevant for *P. flammea*, as LA also exhibited the strongest phylogenetic signal among co‐occurring species along the same elevational gradient (Chaves *et al*., in prep), highlighting its evolutionary role in the expansion of *P. flammea* across heterogeneous environments.

### Seeds thermal requirements

Seed thermal performances in *P. flammea* reflect adaptation to both elevation and local HDD. Notably, HDD is highest in one of the lowland regions, likely due to the high precipitation rates there (2644.5 mm per year; Hijmans *et al*., 2005), which help reduce daily maximum temperatures (Ali & Mishra, 2017; Abatzoglou *et al*., 2022). Highland seeds showed higher cardinal temperatures and required less heat accumulation for germination compared with lowland seeds. Indeed, species from higher elevations tend to exhibit warmer germination preferences, likely as a strategy to avoid frost damage (e.g. Fernández‐Pa*scual et* al., 2017). However, upper (*T*_c_) and lower (*T*_b_) thermal thresholds responded to distinct environmental drivers. *T*_c_ and *T*_o_ (thermal optimum) values were more strongly associated with HDD than with elevation, with seeds from cooler regions (high HDD) reaching *T*_c_ values up to 8°C higher than those from warmer areas (low HDD). By contrast, *T*_b_ was closely related to elevation and declined with increasing germination degree days (GDD). These patterns indicate that germination strategies depend on elevation. Highland populations show an opportunistic strategy with rapid germination (low GDD) during short favorable windows (warm temperatures), while avoiding seed germination during cold periods by raising basal thermal limits. By contrast, lowland populations reduce the risk of germination in short overheating periods by lowering upper germination thermal limits and increasing heat demand for germination (high GDD; Donohue *et al*., 2010; Picciau *et al*., 2019; Lamichhane, 2021; Maleki *et al*., 2024). These findings suggest that regeneration traits are finely tuned to local climates, reinforcing their role in the broad thermal adaptation of *P. flammea*.

Overall, the thermal requirements of *P. flammea* seeds closely mirror the thermal strategies of plants. In particular, the strong correlation between heat tolerance and related leaf traits with seed thermal traits, observed in both field and common‐garden conditions, suggests coordinated adaptation to elevation across life stages. Decreasing elevation led to reductions in thermal limits, considering both photochemical tolerance and seed germination limits, but simultaneously increased heat demand for seed germination (high GDD) and promoted leaf traits related to water saving and thermal regulation, such as LA, SI, and stomatal and trichome densities. Indeed, leaves with higher water content have greater specific heat capacity, which slows the rate of temperature increase (Schymanski *et al*., 2013; Marcelis *et al*., 2025). Higher water storage is also essential for leaf thermal stabilization and enables effective leaf cooling (Aparecido *et al*., 2020; Cook *et al*., 2021; Gräf *et al*., 2021; Luo *et al*., 2021; Posch *et al*., 2024).

In this context, the low variance in heat tolerance observed here and reported in other studies (Cuesta *et al*., 2020; Lancaster & Humphreys, 2020; Chaves *et al*., 2025) likely reflects strong canalization through morphological traits linked to complementary ecological strategies, such as drought avoidance. Rather than indicating that populations are universally close to lethal thermal thresholds under current warming, these results show that heat resistance emerges from integrated suites of physiological tolerance, morphological buffering, and seed‐level avoidance strategies. Consequently, evaluating plant responses to climate change requires consideration of coordinated life‐history and trait strategies, rather than reliance on thermal tolerance metrics alone.

### Conclusion

The coherence of thermal strategies underscores elevation as a key selective driver in shaping the thermal niches of plants. In *P. flammea*, this is expressed as a balance between canalized heat‐avoidance traits and highly plastic cold‐tolerance responses, supporting distribution across a wide elevation gradient. Importantly, these strategies operate across distinct stages, emphasizing that selection acts on the entire life cycle and may influence montane range shifts. Integrating seed‐level thermal traits with plant physiology could substantially improve predictions of population resilience to climate warming. Finally, unraveling these adaptive and plastic traits, including CGV, is essential for mechanistic investigations of ecological thermal strategies.

## Competing interests

None declared.

## Author contributions

CJNC, CP‐S, DE‐E and KJF contributed to the conceptualization. CJNC, DUT, IVL‐S, JPSPB, HV‐B, PA, TCC, MMT, GPS, VdAK, WLdS, LNG and KTS contributed to the data acquisition and methodology. CP‐S, JLSM, RVB and KJF contributed to the resources. CJNC contributed to the formal analysis and original Draft. CJNC and CP‐S contributed to the funding acquisition. CP‐S, KJF and DE‐E supervised the study. CJNC, DUT, IVL‐S, JPSPB, HV‐B, PA, TCC, MMT, GPS, VdAK, WLdS, LNG, KTS, CP‐S, JLSM, DE‐E, RVB and KJF contributed to the review and editing.

## Disclaimer

The New Phytologist Foundation remains neutral with regard to jurisdictional claims in maps and in any institutional affiliations.

## Supporting information


**Fig. S1** Locations of sampled *Pitcairnia flammea* populations included in this study.
**Fig. S2** Schematic representation and detailed layout of the thermal gradient system used to assess the thermal behavior of *Pitcairnia flammea* seeds.
**Fig. S3** Linear relationships between thermal tolerance and leaf traits of *Pitcairnia flammea* individuals with annual mean temperature and heating degree days.
**Fig. S4** Germination rate of seeds from *Pitcairnia flammea* populations distributed along an elevation gradient, shown across different temperatures (from 10 to 35°C).
**Fig. S5** Relationship between the number of days required for 50% of seeds to germinate (*D*_50_) in each *Pitcairnia flammea* population across the tested temperature range (10–35°C).
Please note: Wiley is not responsible for the content or functionality of any Supporting Information supplied by the authors. Any queries (other than missing material) should be directed to the *New Phytologist* Central Office.

## Data Availability

All data supporting the findings of this study have been deposited in the Zenodo repository. The dataset can be accessed at doi: 10.5281/zenodo.17127283.
