# Supplementary material for: Canalized to heat, plastic to cold: adaptive coordination of leaf and seed strategies in populations spanning an elevational gradient
Source: New Phytol. 2026 Jan 11;250(2):873–84. doi: 10.1111/nph.70912 (PMC13001005; doi:10.1111/nph.70912)
Supplement: Supplementary file 1 — Fig. S1 Locations of sampled Pitcairnia flammea populations included in this study. Fig. S2 Schematic representation and detailed layout of the thermal gradient system used to assess the thermal behavior of Pitcairnia flammea seeds. Fig. S3 Linear relationships between thermal tolerance and leaf traits of Pitcairnia flammea individuals with annual mean temperature and heating degree days. Fig. S4 Germination rate of seeds from Pitcairnia flammea populations distributed along an elevation gradient, shown across different temperatures (from 10 to 35°C). Fig. S5 Relationship between the number of days required for 50% of seeds to germinate (D 50) in each Pitcairnia flammea population across the tested temperature range (10–35°C). Please note: Wiley is not responsible for the content or functionality of any Supporting Information supplied by the authors. Any queries (other than missing material) should be directed to the New Phytologist Central Office. [file NPH-250-873-s001.docx]

**Supplementary material**

**Title:**

**Canalized to Heat, Plastic to Cold: Adaptive Coordination of Leaf and Seed Strategies in Populations Spanning an Elevational Gradient**

**Author names:**Cleber J. N. CHAVES^1,2^*, Danilo U. TAVARES¹, Isabella V. LEMOS-SILVA¹, João P. S. P. BENTO^3^, Henrique Vilela da Mata BIANCHINI¹, Paulo AECYO¹, Tami C. CACOSSI^1^, Marília M. TAVARES^1^, Gabriel P. SABINO^1^, Vitor de A. KAMIMURA^1^, Wagner L. dos SANTOS^1^, Lucas N. GONÇALVES^4^, Karina T. SILVA^3^, Juliana L. S. MAYER^3^, Rafael V. RIBEIRO^5^, Diego ESCOBAR-ESCOBAR^6^, Kenneth J. FEELEY^2,7^, Clarisse PALMA-SILVA^1^


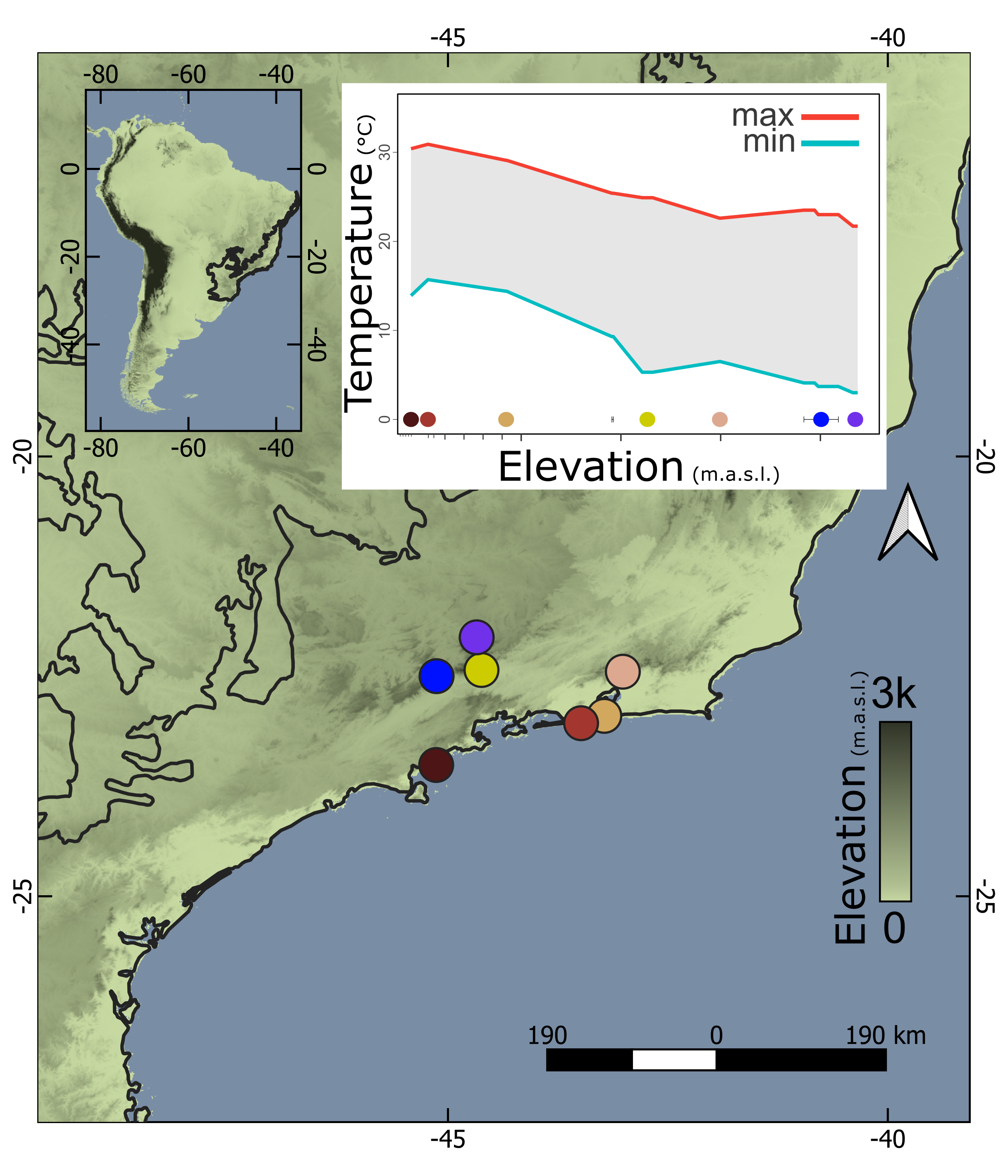


**Figure S1. Locations of sampled *P. flammea* populations included in this study.** The inset details thermal variation (maximum and minimum temperatures) across the elevation gradient.

**
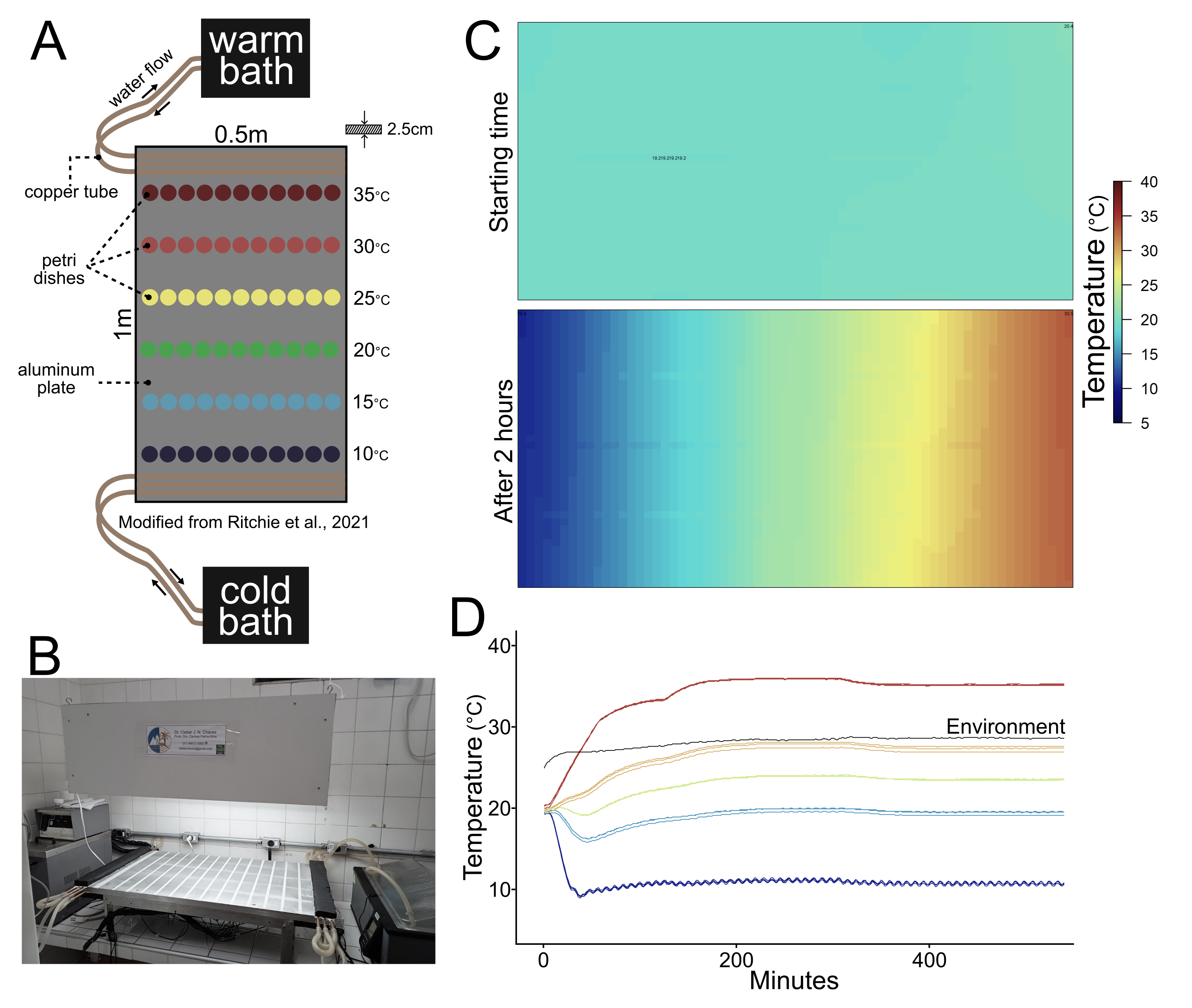
**

**Figure S2. Schematic representation and detailed layout of the thermal gradient system used to assess the thermal behavior of *P. flammea* seeds (A).** In panel B, a photograph of the apparatus is provided, showcasing the thermal gradient plate and the constant light box (opened for photodocumentation) used during the experiment. Panel C illustrates the thermal model of the plate, featuring 15 thermal sensors strategically distributed across the aluminum surface. The plot shows the temperatures at both the starting time and after two hours, offering a visual representation of the temperature distribution. Panel D displays the temperature over time, comparing the plate’s internal temperature changes with the external (ambient) temperature, demonstrating how the thermal gradient stabilizes. Each color represents the position of the 15 sensors, ranging from those closest to the warm bath to those nearer the cold bath. The sensors are organized into five columns, equally spaced along the plate’s length, and three rows, equally spaced along its width.


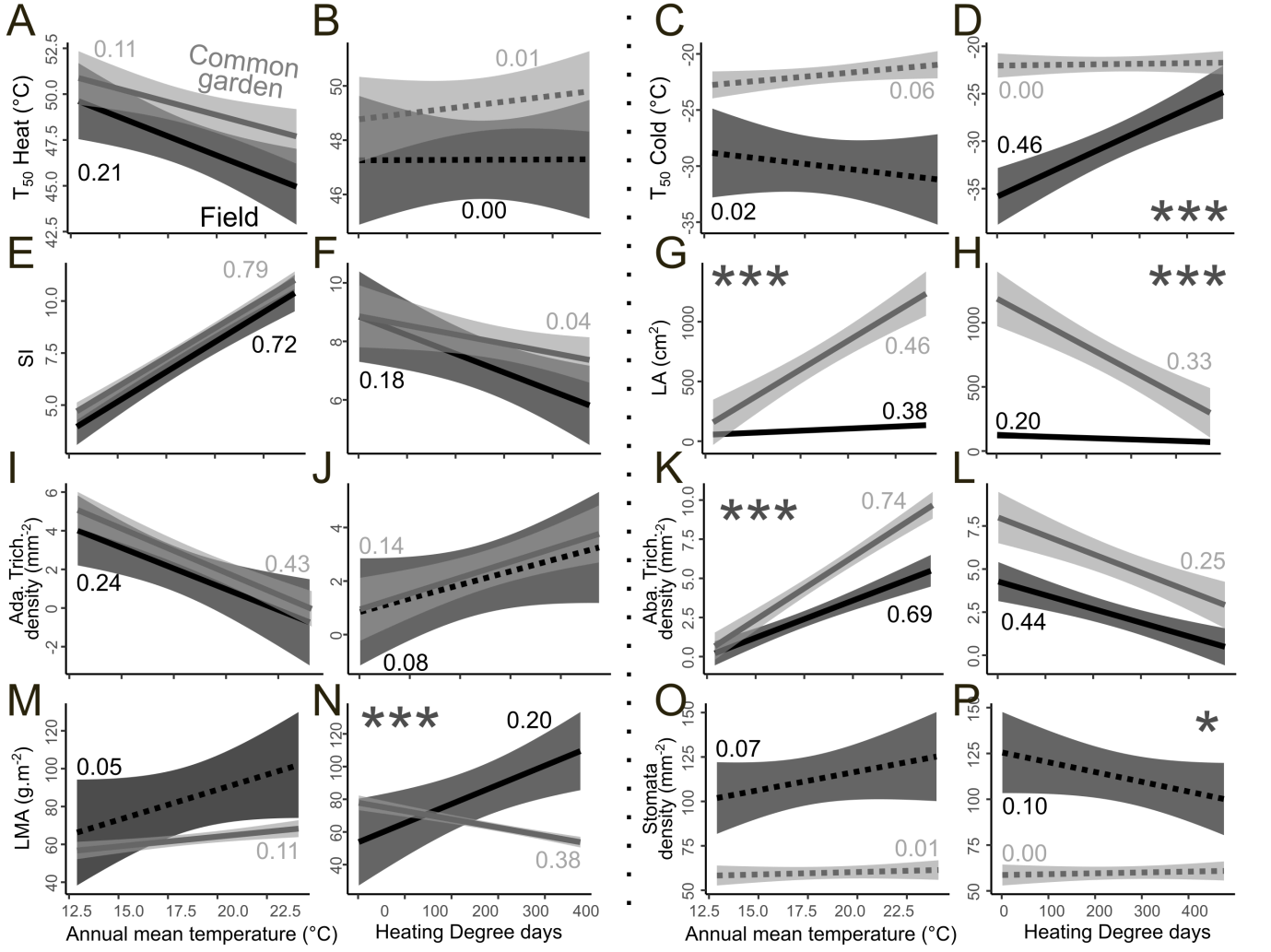


**Figure S3. Linear relationships between thermal tolerance and leaf traits of *P. flammea* individuals with annual mean temperature and heating degree days (HDD).** Black and gray lines (with shaded 95% confidence intervals) represent field-sampled and common-garden plants, respectively. Numbers indicate the R² values of the regressions. Significant differences between slopes are denoted by * p < 0.05, ** p < 0.01, and *** p < 0.001. Dashed lines indicate no significant relationships. T_50_ Heat and Cold: maximum and minimum temperature, specifically causing a 50% decline in chlorophyll F_v_/F_m_. LMA: leaf mass per area; LA: leaf area; SI: succulence index. Stomata: abaxial stomata density. Ada. and Aba. Trich.: adaxial and abaxial trichome densities.


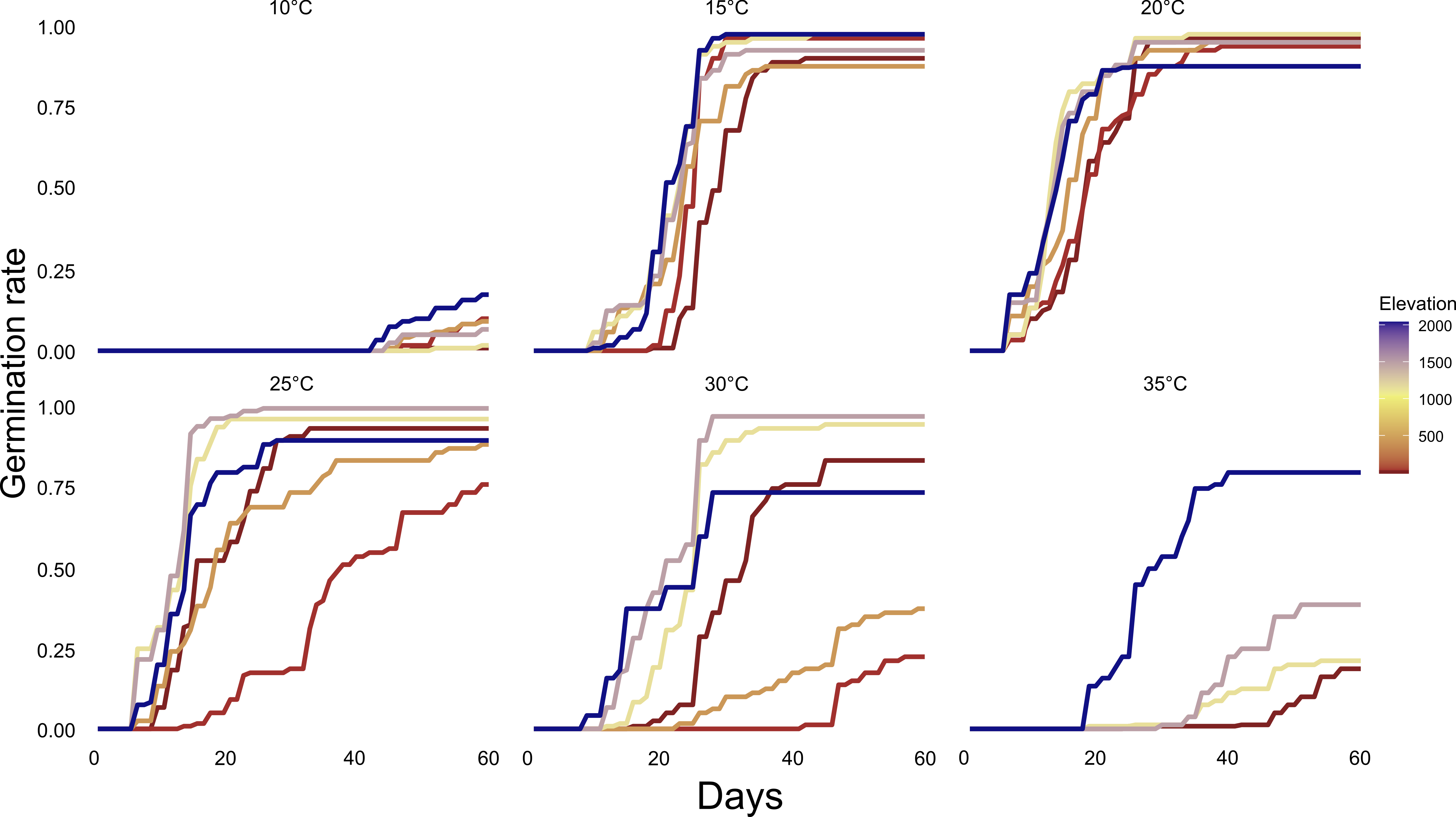


**Figure S4. Germination rate of seeds from *P. flammea* populations distributed along an elevation gradient, shown across different temperatures (from 10 to 35°C).**


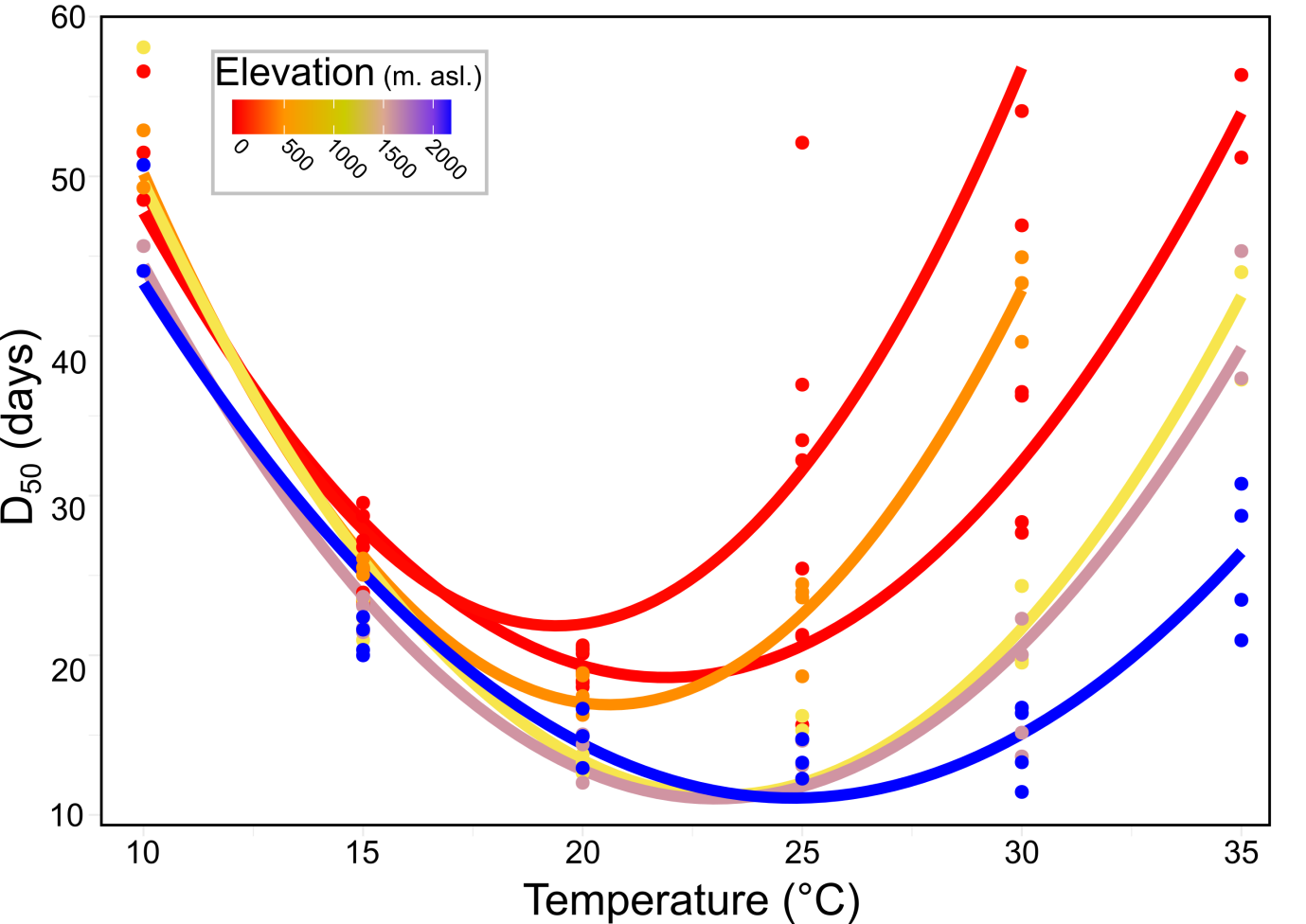


**Figure S5. Relationship between the number of days required for 50% of seeds to germinate (D_50_) in each *P. flammea* population across the tested temperature range (10–35 °C).**
